# Developing a Web-Based Cost Assessment Tool for Colorectal Cancer Screening Programs

**DOI:** 10.5888/pcd16.180336

**Published:** 2019-05-02

**Authors:** Sonja Hoover, Sujha Subramanian, Florence Tangka

**Affiliations:** 1 RTI International, Waltham, Massachusetts; 2 Division of Cancer Prevention and Control, Centers for Disease Control and Prevention, Atlanta, Georgia

## Abstract

**Introduction:**

We developed a web-based cost assessment tool (CAT) to collect cost data as an improvement from a desktop instrument to perform economic evaluations of the Centers for Disease Control and Prevention’s (CDC’s) Colorectal Cancer Control Program (CRCCP) grantees. We describe the development of the web-based CAT, evaluate the quality of the data obtained, and discuss lessons learned.

**Methods:**

We developed and refined a web-based CAT to collect 5 years (2009–2014) of cost data from 29 CRCCP grantees. We analyzed funding distribution; costs by budget categories; distribution of costs related to screening promotion, screening provision, and overarching activities; and reporting of screenings for grantees that received funding from non-CDC sources compared with those grantees that did not.

**Results:**

CDC provided 85.6% of the resources for the CRCCP, with smaller amounts from in-kind contributions (7.8%), and funding from other sources (6.6%) (eg, state funding). Grantees allocated, on average, 95% of their expenditures to specific program activities and 5% to other activities. Some non-CDC funds were used to provide screening tests to additional people, and these additional screens were captured in the CAT.

**Conclusion:**

A web-based tool can be successfully used to collect cost data on expenditures associated with CRCCP activities. Areas for future refinement include how to collect and allocate dollars from other sources in addition to CDC dollars.

**Summary**

**What is already known on this topic?**

Centers for Disease Control and Prevention and RTI International designed a web-based cost assessment tool (CAT) to collect cost and resource data. The design of the CAT was based on published methods of collecting cost data for program evaluation.

**What is added by this report?**

We describe the development of the web-based CAT, evaluate the quality of the data obtained, and discuss lessons learned. We found that grantees were successfully able to collect and report cost data across years by using the web-based CAT.

**What are the implications for public health practice?**

Data on activity-based expenditures and funding sources, collected using the web-based CAT, are essential in planning for the allocation of limited health care resources.

## Introduction

Colorectal cancer (CRC) screening can detect early-stage CRC and adenomatous polyps (1,2). However, CRC screening remains low; only 67.3% of adults aged 50 to 75 years in the United States received CRC screening that was consistent with US Preventive Services Task Force recommendations (3). To explore the feasibility of a CRC screening program for the underserved US population, the Centers for Disease Control and Prevention (CDC) established the Colorectal Cancer Screening Demonstration Program (Demo), conducted from 2005 through 2009. In 2009, CDC modified and expanded efforts to promote and provide CRC screening to 29 states and tribal organizations through the Colorectal Cancer Control Program (Program 1), which was conducted from 2009 through 2014) (4).

CDC and RTI International conducted economic evaluations as part of the Demo and Program 1. To help improve cost evaluation of CRC screening, we developed a cost assessment tool (CAT) to collect cost data and to perform economic evaluations. Cost assessments allow program planners and policy makers to determine optimal allocation of limited health care resources, identify the most efficient approach to implementing screening programs, and assess annual budget implications (5). However, cost assessment is a challenge for CRC programs because funds may come from many sources, different tests may be used (eg, colonoscopy vs stool tests), grantees may choose different screening promotion activities, and grantees may have different relationships with state and local public health organizations or with contractors. We focus on the CAT for Program 1 and describe lessons learned from designing and implementing the CAT to improve future data collection efforts.

## Methods

The design of the web-based CAT for Program 1 (OMB no. 0920–0745) was based on published methods of collecting cost data for program evaluation (5–14). We collected data from a programmatic perspective on all funding sources of the grantees, including federal, nonfederal (eg, state or national organizations), and in-kind. We then asked grantees to collect cost data on activities relevant to the program in 5 budget categories: labor; contracts, materials, and supplies; screening and diagnostic services for each screening test provided; consultants; and administration. In the Program 1 CAT, all budget categories were allocated to specific program activities. We asked grantees to provide costs on screening promotion and provision activities. We also had a category of overarching activities, which included activities to support both screening promotion and provision activities, such as program management and quality assurance (Appendix).

### Data collection procedures

For the Demo, we initially collected all resource use data via a Microsoft Excel-based tool. However, in 2009 we piloted a web-based tool. The web-based tool allowed us to embed data checks within the Program 1 CAT; therefore, fewer mistakes would be made during data entry and the quality of the data received would be improved. Examples of the embedded checks included asking grantees to allocate at least 95% of the total amount of annual funding to specific program activities in their reporting. By using an algorithm indicating that total allocation to spending activities was equal to or greater than 95% of total funding, the grantee could submit the CAT; if total allocation was less than 95%, the grantee needed to revise inputs. Grantees also had to confirm that 100% of staff time spent was allocated to specific activities and that the amount of funding received and the amount of carryover funding from previous years were accurate. If any of these checks failed, the grantee would need to review and revise inputs before submitting. Because of the ease of use of the web-based tool and the success of embedded checks, we implemented the web-based tool for Program 1.

In the Demo version of the CAT, we had an overall “other” category for activities that were not easily placed in existing categories. The activities that were placed in this “other” category were activities where grantees received lump-sum amounts that could not be easily divided among existing activities, or activities that were not included in the existing list. In the Program 1 web-based CAT, we added 2 “other” categories: an “other” category specifically for screening promotion activities, and an “other” category specifically for screening provision activities. In analyzing the second year of Program 1 CAT data, we found commonalities in activities that were included in the “other” categories, leading us to add more activities to the CAT, including patient navigation for both screening promotion and screening provision components and mass media for screening promotion activities.

In addition to collecting Program 1 cost data, we collected data on the number of screenings conducted by the grantee. We asked grantees to report on total number of individuals screened, screening tests performed by test type, follow-up colonoscopies, adenomatous polyps/lesions detected, and cancers detected. We also asked grantees to report total number of people previously diagnosed with CRC who were undergoing follow-up surveillance for recurrence or development of CRC and total number of people enrolled in insurance programs. We used this data to supplement the Colorectal Cancer Clinical Data Elements (CCDEs) that Program 1 grantees provided CDC. While the CCDEs collected data only on screenings funded by CDC, the Program 1 CAT collected the same information for all screenings facilitated by the grantee regardless of the source of funding.

To maintain systematic and standardized data collection, we provided all grantees with a data user’s guide and provided technical assistance via teleconferences and email. We hosted webinars about how to collect and input data using the Program 1 web-based CAT. The information in the Program 1 CAT was collected retrospectively; however, to improve the accuracy of the data, grantees were encouraged to track and log information required prospectively when feasible. Cost data were collected and analyzed on an annual basis for 5 years (2009–2014). In the first year of Program 1, we collected data from 26 grantees; in years 2 through 5, we collected data from 29 grantees annually.

### Data quality assessment and analysis

On completion of the annual submission of the Program 1 CAT, we conducted data quality checks. We confirmed that data were entered into each of the broad categories of personnel, contracts, screening provision, and administration/overhead. If no costs were reported in any of these categories, we followed up with grantees to understand why. When funding amounts were reported for screening provision, we verified that screening numbers were also provided in the tool (or to CDC in year 5). Each year, before releasing the Program 1 annual CAT summary data to grantees, CDC also compared the data reported in the CAT with information in the fiscal database on approved, expended, and carryover funds. We generally found high levels of concordance.

To analyze the Program 1 CAT, we calculated staff cost per activity by using salary information and hours spent by staff members on each activity. We also calculated cost of contracts by activity and prepared a summary of cost data for each submission for each grantee’s review and approval. The summary provided grantees with information on the labor and nonlabor cost per activity and costs by budget category and in-kind contributions.

We performed 4 different analyses. First, we examined the distribution of funding from CDC, in-kind, and other sources. This evaluation of funding was essential to identify the extent to which assessments based on CDC funding alone would provide valid results and whether all funding sources needed to be considered. Second, we analyzed the cost by budget categories because certain types of data, such as contracts, tend to be more difficult to allocate to specific activities. Third, we examined the distribution of expenditures for screening promotion activities, screening provision activities, and overarching activities for each year of the program. On the basis of qualitative feedback from grantees, we anticipated that the overarching component would be high in year 1 because a large proportion of resources was initially allocated to planning activities; similarly, screening provision costs would be low because of the contracts that needed to be executed before initiating screenings of eligible individuals. Fourth, we evaluated the percentage of total expenditures that was not allocated to specific activities by year (ie, reported as miscellaneous “other” activity) and summarized the “other” activity category across all program years. As a quality measure, we required that at least 95% of the total funding be allocated to specific budget categories.

We compared the number of people screened based on data from both the Program 1 CAT and from the CCDEs. For grantees reporting other funding sources in addition to CDC dollars and where the other sources were earmarked for screening provision, we anticipated higher reported numbers of people screened. To reduce the reporting burden, in year 5 we did not require grantees to report the number of people screened if they received only CDC funding. We asked only grantees who received funds from non-CDC sources to report screens in the Program 1 CAT and used the screens reported in the CCDEs for grantees who reported only CDC funding (some of these grantees continued to voluntarily report the screens performed in the CAT). Because we could not verify additional funding for all grantees for each year in sufficient detail, we excluded selected grantees from this comparative analysis. We excluded grantees if they had not yet begun screening (generally during year 1) and if they reported extra funding inconsistently across years (eg, if a grantee reported extra funding in years 1, 3, and 4 but not in 2, they were excluded because screens sometimes overlap across years, and we could not ensure accuracy). Overall, we had a total of 45 program years with only CDC funds for screening, and 57 program-years with other funding for screening.

## Results

**Figure 1:**
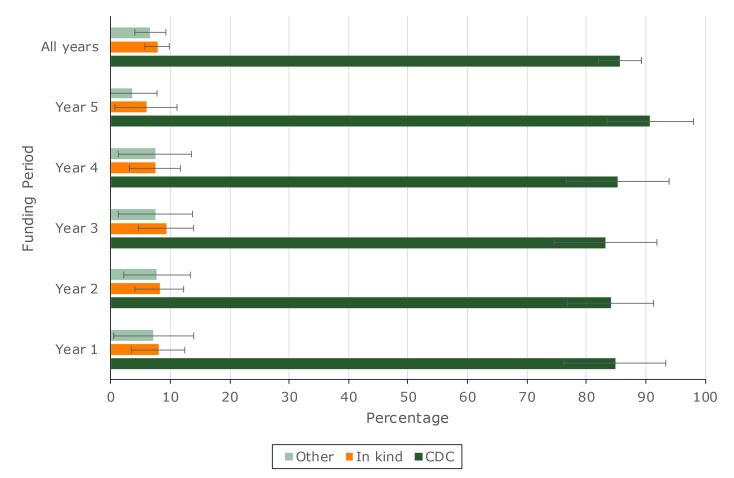
Percentage distribution of funding sources, by year, Colorectal Cancer Control Program, 2009–2014. Error bars indicate confidence intervals.

On average, most funding (85.6%) was from CDC (Figure 1). A smaller proportion of grantees indicated that they received in-kind contributions (7.8%) or funding from other sources (6.6%). The total amount from all sources equaled $148,016,341.

**Table:** 

Source	% (95% Confidence Interval)					
	Year 1	Year 2	Year 3	Year 4	Year 5	All Years
Other	7.2 (0.5–13.9)	7.7 (2.1–13.3)	7.5 (1.2–13.8)	7.4 (1.2–13.6)	3.5 (0–7.7)	6.6 (4.0–9.2)
In kind	8.0 (3.5–12.5)	8.2 (4.1–12.3)	9.3 (4.6–14.0)	7.4 (3.1–11.7)	5.9 (0.7–11.1)	7.8 (5.8–9.8)
CDC	84.8 (76.3–93.3)	84.1 (76.8–91.4)	83.2 (74.5–91.9)	85.3 (76.6–94.0)	90.7 (83.4–98.0)	85.6 (82.0–89.2)

In year 1, most costs (43.8%) were for overarching activities, which decreased to 37.2% by year 5 (Table 1). Screening promotion activities costs accounted for one-third of cost in year 1 and decreased to 27.5% by year 5. Screening provision activities comprised the smallest percentage of total costs in year 1 and ranged from 34% to 39% for years 2 to 5. The total amount by year ranged from $22,612,125 in year 1 to $33,037,756 in year 2.

**Table 1:** Distribution of Total Costs, by Year and Activity, Colorectal Cancer Control Program, 2009–2014

Activity	Year 1	Year 2	Year 3	Year 4	Year 5	All Years
Screening promotion activities, % (95% CI)	33.1 (25.5–40.8)	26.8 (20.5–33.2)	26.7 (21.1–32.3)	24.2 (18.5–29.9)	27.5 (21.8–33.3)	27.6 (24.8–30.3)
Screening provision activities, % (95% CI)	23.0 (16.9–29.2)	33.7 (27.4–40.0)	33.8 (28.8–38.8)	38.6 (33.0–44.1)	35.2 (29.6–40.9)	33.1 (30.4–35.7)
Overarching activities, % (95% CI)	43.8 (36.1–51.6)	39.5 (31.7–47.3)	39.4 (33.6–45.3)	37.3 (31.5–43.0)	37.2 (31.7–42.7)	39.4 (36.4–42.3)
Total costs, $	22,612,125	33,037,756	32,247,955	31,439,050	28,679,456	148,016,341

Abbreviation: CI, confidence interval.

**Figure 2:**
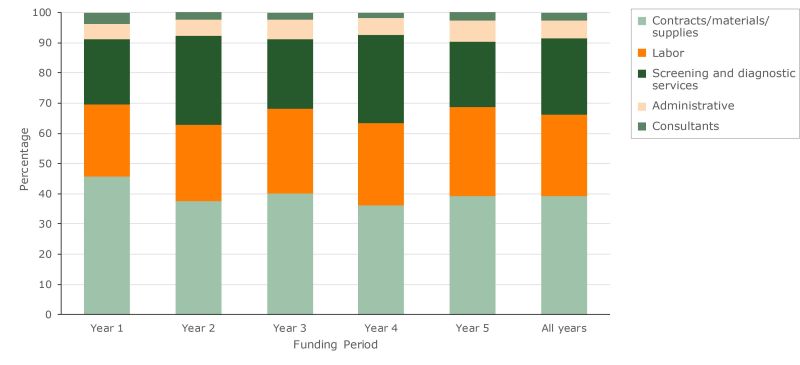
Percentage distribution of total cost by budget category, Colorectal Cancer Control Program, 2009–2014.

The largest total cost category in all years was contracts, materials, and supplies (Figure 2). This category was intended primarily for nonclinical services and averaged 39.4% across the years. Contracts generally comprised the largest part of these costs, while materials and supplies accounted for a smaller portion. The lowest costs were in the consultants category: grantees reported using less than 4% of their funding for consultants in any year.

**Table:** 

Category	Year 1	Year 2	Year 3	Year 4	Year 5	All Years
Contracts, materials, and supplies	45.7	37.6	40.0	36.3	39.3	39.4
Labor	24.0	25.3	28.3	27.1	29.4	26.9
Screening and diagnostic services	21.4	29.3	22.9	29.1	21.7	25.2
Administrative	5.1	5.3	6.4	5.7	6.9	5.9
Consultants	3.8	2.6	2.4	1.7	2.8	2.6

Overall, grantees were able to allocate more than 95% of their costs to specific program activities. Across years, grantees were unable to allocate approximately 2.4% of costs among promotion activities, 0.8% of costs among provision activities, and 1.8% of costs among other activities (Table 2).

**Table 2:** Percentage of Total Costs Allocated to “Other” Cost Category^a^, Colorectal Cancer Control Program, 2009–2014

Activity	Year 1 (95% CI)	Year 2 (95% CI)	Year 3 (95% CI)	Year 4 (95% CI)	Year 5 (95% CI)	All years (95% CI)
Promotion: other screening promotion activities	2.7 (1.2–4.2)	3.5 (1.1 to 6.0)	2.7 (0.9 to 4.5)	1.0 (0.0 to 2.0)	2.2 (0.4 to 4.0)	2.4 (1.6 to 3.2)
Provision: other screening provision activities	0.9 (−0.6 to 2.4)	0.6 (−0.1 to 1.3)	1.2 (0.0 to 2.4)	0.2 (−0.1 to 0.4)	0.9 (0.3 to 1.5)	0.8 (0.3 to 1.2)
Other activities	1.9 (0.9 to 2.8)	1.3 (0.1 to 2.6)	1.1 (−0.1 to 2.3)	2.2 (0.6 to 3.8)	2.7 (1.6 to 3.8)	1.8 (1.3 to 2.4)

Abbreviation: CI, confidence interval.

a Activities in the “other” category were combined activities that received lump-sum amounts that could not be easily divided among existing activities or were activities that were not included in the list of activities provided to grantees.

Grantees who received funding from other sources reported higher average screening numbers in the Program 1 CAT compared with the CCDEs (Table 3). Across all years, grantees with additional funding reported an average absolute difference of 976 individuals screened compared with what was reported in the CCDEs. Grantees that did not report additional funding reported similar screening numbers as in the Program 1 CAT and the CCDEs with an average absolute difference across all years of 6.

**Table 3:** Comparison of Number of People Screened^a^ Reported in Program Cost Assessment Tool (CAT) and CDC Colorectal Cancer Clinical Data Elements (CCDEs)^b^

Category (No. of Grantees)	Average Absolute Difference Between Reporting Methods					
	Year 1	Year 2	Year 3	Year 4	Year 5^c^	All Years
Grantees reporting extra funding (57)^d^	1,284	1,032	689	978	978	976
Grantees not reporting extra funding (45)^e^	3	15	5	3	1	6

a Number of people who reported having a colorectal cancer screen using either fecal occult blood test, fecal immunochemical test, sigmoidoscopy, or colonoscopy.

b Average absolute difference was calculated by averaging the absolute differences between the number of individuals screened and reported in the CAT and the number screened and reported in the CCDEs. The total number of screens reported on the CAT was 76,297 (ranging from 16 to 9,762) and on the CCDEs was 20,997 (ranging from 16 to 1,460).

c Exclusions are different than in previous years because in year 5 we allowed grantees to defer to CCDEs numbers instead of reporting screening numbers on the CAT.

d The number of grantees included who reported extra funding was 8, 13, 14, 12, and 10 in years 1, 2, 3, 4, and 5, respectively.

e The number of grantees included who did not report extra funding was 10, 9, 9, 11, and 6 in years 1, 2, 3, 4, and 5, respectively.

## Discussion

We described how we developed and used a standardized cost data collection instrument to support an economic evaluation of grantees participating in Program 1. We collected data from grantees annually for 5 years. We found that, in addition to CDC, in-kind cost contributions and funding from other sources were important sources of assistance to the programs, although not all grantees indicated that they were recipients of additional contributions. Among those who did, many did not receive or report additional contributions consistently. This may account for the slight variation in distribution of funding sources across years, particularly in the last year of the program. To collect this data more accurately in the future, grantees need to be provided guidance on how to collect this information.

Spending for overarching activities, those that supported both screening promotion and screening provision activities, were a significant portion of grantees’ expenditures, particularly in the early years. This was not surprising because programs were in their start-up phases and had not yet begun in earnest to promote or provide CRC screening. Grantees needed time, for example, to hire their staff and form partnerships to make the programs viable. As the programs were implemented, the proportion of funding used for these overarching activities generally decreased, as expected.

All grantees reported cost data by budget category, and more than half of expenditures was allocated to labor and to screening and diagnostic services. On the basis of previous experience in using a cost tool (to collect resource use data for the National Breast and Cervical Cancer Early Detection Program), labor and clinical service costs are typically captured accurately (15). However, allocating contracts expenditures can be more difficult to accomplish systematically because contract funds are provided to partners who often do not report details on the activities performed to the grantees. Future studies can be designed to collect additional information in a consistent manner from partner organizations to increase the completeness and quality of the activity-based cost assignment.

We found that grantees were able to collect and assign most of their costs to activities conducted during the program years. All grantees were able to allocate 95% or more of their funding to specific activities in the Program 1 CAT. To achieve this detailed reporting, we found numerous processes critical. First, it was important to solicit grantee input in formulating the activity listing in the CAT. Although there was a focus on evidence-based interventions recommended in *The Guide to Community Preventive Services* by the Community Preventive Services Task Force (such as client reminders and small media), grantees also conducted screening promotion activities that were not evidence-based interventions (eg, patient navigation, professional training) that we ultimately included in the Program 1 CAT (16). Second, grantee input during the design of the web-based CAT was invaluable in terms of creating a user-friendly tool. The web-based CAT had embedded checks to ensure efficiency in collection of high-quality data, which included a 1-step review and finalization process before submission. Third, we had a dedicated staff person provide technical assistance to grantees via telephone or email. We also drafted a detailed user’s guide that contained definitions of activities and step-by-step instructions on how to enter data.

We found it was essential on the Program 1 CAT to solicit from grantees the number of people screened for CRC and the number of screens conducted. Nearly 15% of funding was from sources other than CDC, and much of this funding was allocated to screening provision. Grantees with additional funding sources were able to conduct more screens than grantees who did not report added funding. Had we not asked for this information, we would have substantially underestimated the number of people screened. With the complete screening information available in the CAT, we were able to perform a comprehensive assessment of the cost per screen to inform future program planning (15,17).

We encountered numerous limitations in analyzing cost data collected by grantees. Although we collected 5 years of data from grantees, the ultimate sample size was small for each period (26 grantees in year 1, 29 grantees in years 2–5). We attempted through trainings and the user’s guide to define the program activities for the grantees and designate how to allocate time. Although in most cases we were able to attribute most of the costs to program activities, some inconsistencies are likely in how grantees ultimately defined the activities. We also anticipated recall bias related to time inputs, although we tried to alleviate that through training and by encouraging tracking of data prospectively. Lastly, some grantees were unable to disaggregate contracts and other inputs into activities and could only report them as a lump sum. These were allocated into the “other” categories.

We provide details on the methods used to collect data on activity-based expenditures and funding sources. These data are necessary to plan for optimal use of resources, and the additional details in these data are advantageous compared with previously existing resources such as budget or funding information. Although the focus of the CAT is specifically on cost and resource collection for the Demo and for Program 1, the CAT can be customized. For example, the CAT has been used with success in estimating cost and resource use for both national and international cancer registries and for other cancer screening programs (18–26). On the basis of lessons learned from this 5-year data collection effort, the CAT was redesigned and tailored for each individual grantee for Program 2 (2015–2020), which is the next iteration of the Program.

## Appendix Description of Colorectal Cancer Control Program Activities

**Table:**

Program Activity	Description
**1. Screening promotion activities**	
1a) Client reminders	Reminders include letters, postcards, emails, or phone calls to alert patients that it is time for their cancer screening. Some reminders note only that the test is due, while others include facts about the screening or offer to help set up an appointment in addition to including a reminder that the test is due.
1b) Small media	Small media include videos and printed materials such as letters, brochures, and newsletters. These materials can be used to inform and motivate people to be screened for cancer. They can provide information tailored to specific individuals or targeted to general audiences.
1c) Mass media	Mass media include radio, television, billboards, magazines, newspapers, public service announcements, and advertisements.
1d) Outreach/incentives/patient education	Outreach/incentives/education include outreach activities, such as attendance and activities at health fairs, costs of incentives to patients to participate in programs, and activities related to patient education.
1e) Provider assessment and feedback	These interventions assess how often providers offer or deliver screening to clients (assessment) and then give providers information about their performance (feedback). The feedback may describe the performance of an individual provider or of a group of providers (eg, mean performance for a practice). The performance may be compared with a goal or standard.
1f) Provider reminders	Reminders inform health care providers that it is time for a client’s cancer screening test (called a “reminder”) or that the client is overdue for screening (called a “recall”). The reminders can be provided in different ways, such as flagging client charts, building provider reminders into electronic medical record systems or provider office appointment systems, or by email to the provider.
1g) Reduction in structural barriers	Many structural barriers (eg, distance from screening location, limited hours of operation, lack of day care for children, language and cultural factors) can make it difficult for people to seek screening for cancer. Interventions designed to reduce these barriers may facilitate access by Reducing time or distance between service delivery settings and target populations Modifying hours of service to meet client needs Offering services in alternative or nonclinical settings Eliminating or simplifying administrative procedures and other obstacles (eg, revising clinic flow procedures, adopting electronic medical records systems) We are not asking about patient navigation services here; patient navigation for screening promotion and screening provision is covered elsewhere in the Cost Assessment Tool.
1h) Patient navigation and support	Establishing a patient support system or using patient navigators can ensure that appropriate screening, diagnostic, and treatment services are received in a timely manner. Some programs may refer to this as case management. Some roles of the patient navigator include Assisting with scheduling appointments, transportation, or dependent care Providing patient education about colorectal cancer screening and testing modalities regarding screening (eg, rationale, importance, bowel prep) Reminding patients about their colonoscopy appointment or returning their fecal occult blood test/fecal immunochemical test kits Providing peer support to help with cultural or emotional concerns (eg, allay fears)
1i) Reduction in out-of-pocket costs	Interventions could include reducing the costs of the screening tests, providing vouchers, reimbursing clients or clinics, and/or reducing health insurance costs.
1j) Enrolling in insurance programs	Assistance is provided to individuals eligible for Medicaid or other insurance programs to enroll.
1k) Other screening promotion activities (please specify)	Programs report any additional screening promotion activity that is not reportable under the above options and provide a description of the activity.
**2. Screening provision activities**	
2a) Manage provider contracts, billing systems, and other procedures	Manage contract with local physicians and clinics to deliver screening services Monitor administrative billing and reimbursement system
2b) Patient navigation and support (client directed)	Use patient navigators to ensure that timely screening and diagnostic services are provided to clients screened by the program.
2c) Provide screening and diagnostic services	Provide colorectal cancer prescreening, screening, diagnostic follow-up, and surveillance colonoscopy services.
2d) Ensure appropriate treatment of complications and cancers	Develop and execute a plan to obtain treatment services for people diagnosed with cancer or experiencing medical complications.
2e) Other screening provision activities (please specify)	Report any additional screening provision activity that is not reportable under the above options and provide a description of these activities.
**3. Overarching activities**	
3a) Program management	Monitor program performance Manage fiscal system Manage contract with local physicians and clinics to deliver screening services Coordinate administrative-related policies and procedures Manage programmatic/administrative/reporting issues; travel for program meetings Monitor administrative billing and reimbursement system Recruit, hire, and train staff members as required on an ongoing basis Continue to collaborate with Centers for Disease Control and Prevention (CDC)
3b) Quality assurance and professional development	Monitor quality control standards and mechanisms Continually review clinical policies and procedures Educate and train health care professionals
3c) Partnership development and maintenance	Maintain a relationship with the CDC-funded comprehensive cancer control implementation program Maintain partnerships with diverse group of entities
3d) Clinical and cost data collection and tracking	Monitor and provide feedback by using patient data tracking system Collect and report person-level clinical data Collect and report cost data
3e) Program monitoring and evaluation	Collaborate with CDC in the monitoring and evaluating of the overall program Implement program-specific monitoring and evaluation
3f) Other activities	Report any additional activities that are not reportable under the above options and provide a description of these activities.
